# Charting an Equity-Centered Public Health Data System

**DOI:** 10.1089/big.2022.0203

**Published:** 2022-09-06

**Authors:** Alonzo L. Plough

**Affiliations:** Robert Wood Johnson Foundation, Princeton, New Jersey, USA.

Everyone deserves a fair and just opportunity to live a long and healthy life. To achieve this vision, we first must have a public health data system rooted in health equity—a system that reflects the health needs and experiences of all people in America, regardless of where they live, the color of their skin, how much money they make, or how they identify themselves.

Building a public health data system that works for all requires collaboration among all, across sectors as diverse as health care, business, education, housing, transportation—and, yes, big data. That is the driving force behind this special journal issue: to highlight the critical role of the data and tech industry in advancing health equity by helping to create a modern public health data system.

Last year, I worked alongside the National Commission to Transform Public Health Data Systems, a first-of-its kind independent body established by the Robert Wood Johnson Foundation (RWJF) to reimagine how data are collected, shared, and used to advance health equity. The commission's recommendations, released in October 2021, will help shape health information and data architecture and systems for years to come.

During my work with the commission, I was struck not just by the lack of equity inherent in our public health data systems but also by the numerous inequities that have become embedded in them: communities, especially people of color, that are poorly represented by public health data; unexamined assumptions in data algorithms that exacerbate health disparities; and failure to disaggregate data in ways that meaningfully capture the health experiences of historically marginalized populations.

Our current system was simply not designed to meet the needs of our many diverse communities. As a result, we have data that are incomplete, inaccurate, out of date, and fragmented. They do not provide a true picture of health at the community level, which means that policymakers do not have the information they need to target their resources judiciously and effectively.

We have seen how that has worked out throughout the COVID-19 pandemic. Lack of comprehensive, timely, and accurate information prevented public health officials at all levels from identifying and addressing problems with the urgency they required. It took far too long, for example, to recognize the pandemic's disproportionate impacts on Black, Latino, and Indigenous communities, as well as on people in congregate living arrangements.

We can and must do better. We need to build the infrastructure that will produce and leverage next-generation data that are more valid, more meaningful, and more actionable in communities and across sectors working to advance change.

And we need the tech and data sector to help us get there. There are many challenges to address. For example, how can we align data from outside the health care system? Because health is shaped by more factors than medical care—for example, housing, jobs, and education—we need data from multiple systems and sectors to get a complete picture of health at the community level. Although data scientists can be pivotal in helping us to think about how to get that information, we can encourage them to think about how to capture this information with an inclusionary equity lens.

We also need guidance on the kinds of information we should target, and on the best ways to interrogate it, disaggregate it, contextualize it, and make sure it is actionable. How do we bring narrative data into the kind of system we want to build? What are equitable data practices?

We also hope that you in the tech sector will work with community partners to cultivate trusting environments where those closest to the challenges are shaping solutions and local residents feel comfortable sharing their information for the good of public health. This is crucial for building a data system that advances equity, and there is a lot of work to be done here.

As you can see, the challenge here is not just about modernizing the same old public health data systems that have been around for 20 years and more. It is about building something fundamentally new: a system that helps ensure that everyone can achieve and maintain good health.

The articles in this special issue were written with that vision in mind; several of them even informed the commission's deliberations. Each article addresses an issue essential to the challenge of building an equity-focused public health data system:

- Why Equity Matters in Public Health Data. Authors Anita Chandra, Laurie T. Martin, Joie D. Acosta, Christopher Nelson, Douglas Yeung, Nabeel Qureshi, and Tara Blagg explore where and how equity has been lacking in public health data and the implications of considering equity to the tech and data sectors.
- What is Public Health Data? As authors Joie D. Acosta, Anita Chandra, Douglas Yeung, Christopher Nelson, Nabeel Qureshi, Tara Blagg, and Laurie T. Martin explain, good public health data are more than just health data. We need to reimagine the types of data we collect and from where, as well data precision, granularity, timeliness, and more.
- Public Health Data and Special Populations. People of color, women, people with disabilities, and people who are lesbian, gay bisexual trans-gendered queer are among the populations that have been inconsistently represented in public health data over time. This article by authors Tina J. Kauh and Maryam Khojasteh reviews findings for each population, as well as commonalities across populations.
- Public health data interoperability and connectedness. What are challenges to connecting public health data swiftly yet accurately? What gaps need to be filled? How can the data and tech sector help address these issues? These are some of the questions explored in this article by authors Laurie T. Martin, Christopher Nelson, Douglas Yeung, Joie D. Acosta, Nabeel Qureshi, Tara Blagg, and Anita Chandra.
- Integrating Tech and Data Expertise into the Public Health Workforce. This article by authors Laurie T. Martin, Anita Chandra, Christopher Nelson, Douglas Yeung, Joie D. Acosta, Nabeel Qureshi, and Tara Blag envisions what a tech-savvy public health workforce will look like and how it can be achieved through new workforce models, opportunities to expand capacity, and training.

The tech and data industry has a unique opportunity to improve health for all by helping to truly create a 21st century public health data infrastructure rooted in equity and fairness. We hope you will join us in this enterprise.

## Abbreviation Used

RWJF: Robert Wood Johnson Foundation

